# Standards and Regulations for Battery Management Systems in Germany: Review and Improvement Potentials

**DOI:** 10.1002/gch2.202500129

**Published:** 2025-07-29

**Authors:** Dilane Dongmo Tadoum, Franziska Berger, Florian Krause, David Wasylowski, Florian Ringbeck, Weihan Li, Dirk Uwe Sauer

**Affiliations:** ^1^ Chair for Electrochemical Energy Conversion and Storage Systems Institute for Power Electronics and Electrical Drives (ISEA) RWTH Aachen University Campus‐Boulevard 89 52074 Aachen Germany; ^2^ Center for Ageing, Reliability and Lifetime Prediction of Electrochemical and Power Electronic Systems (CARL) RWTH Aachen University Campus‐Boulevard 89 52074 Aachen Germany; ^3^ Juelich Aachen Research Alliance JARA‐Energy Templergraben 55 52056 Aachen Germany; ^4^ Helmholtz Institute Münster (HIMS) IMD‐4, Forschungszentrum Jülich 52425 Jülich Germany

**Keywords:** batteries, battery management system, lithium‐ion batteries, review, standards

## Abstract

Battery performance and safety heavily depend on battery management systems (BMS), which monitor and control them during operation. Given its crucial role, a BMS should meet several requirements in functionality, performance, robustness, and reliability, often defined in standards and regulations. Considering rapid technological advancements in batteries, updating these requirements is essential to reflect growing system complexity. Therefore, this study reviews current standards and regulations for BMSs in Germany, a key player in the global battery sector. It distinguishes between functional and non‐functional, as well as qualitative and quantitative requirements. The review finds that most existing standards focus on qualitative aspects and lack measurable benchmarks, particularly for critical BMS functions like state monitoring and energy management. To address this, this study proposes improvement suggestions and highlights the need for consistent definitions and performance requirements, especially for the state of charge (SoC) and state of health (SoH). It also identifies emerging challenges, such as second‐life batteries, BMS, and cloud BMS as important areas for future standards. By mapping standards to BMS functions and identifying gaps, this work offers valuable guidance for improving BMS performance, interoperability, and safety.

## Graphical abstract

Battery management systems (BMS) are critical for battery performance and safety. This paper reviews current German standards, highlighting gaps in measurable requirements for key BMS functions like SoC, SoH, and thermal management. It proposes improvements and emphasizes future needs, including second‐life use, AI diagnostics, and cloud connectivity, offering guidance for developers, regulators, and researchers to enhance BMS reliability and interoperability.

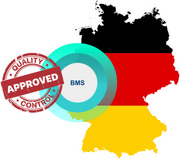

## 1 Introduction

Batteries^[^ ^1^ ^]^ are indispensable today, as they are crucial in various applications, such as electromobility (e.g., electric vehicles, electric scooters) and mobile devices (e.g., mobile phones, laptops, cordless electric tools). However, batteries are sensitive electrochemical systems that, if improperly operated, can drastically lose performance^[^ ^2^, ^3^ ^]^ and pose safety risks.^[^ ^4^ ^]^ This is true for lead‐acid batteries and more advanced battery technologies such as lithium‐ion batteries (LIBs) and high‐temperature batteries. Incidents of electric devices catching fire due to their batteries are numerous, underscoring their potential dangers.^[^ ^5^, ^6^, ^7^, ^8^, ^9^, ^10^, ^11^ ^]^ Therefore, monitoring and controlling batteries while operating is crucial to ensure they remain within their safe operating area (SOA). This important task falls under the responsibility of the battery management system (BMS), also known as the battery management unit (BMU) or battery control unit (BCU). Furthermore, the BMS is also tasked with maximizing the battery's short‐term (power, efficiency, temperature, and safety) and long‐term performance (lifetime). Maximizing the battery lifetime helps to reduce their environmental impact.^[^ ^12^, ^13^, ^14^, ^15^ ^]^

Considering its key role for the battery safety and performance, a failure or malfunction of a BMS can lead to loss of consumer trust, financial losses (e.g., reduced operating time or recall actions^[^ ^16^, ^17^, ^18^, ^19^ ^]^), irreversible battery damage, and even safety hazards. Numerous battery fire incidents have been reported, in which the BMS was partly responsible, as it failed to monitor and control the battery correctly. The role of the BMS in some of these cases is reviewed in Refs. [11, 18, 20]. For instance, Hyundai recalled around 82 000 EVs in 2021 after several battery fire incidents worldwide. According to LG Energy Solutions, a division of the LG Group, which manufactured the batteries, the Hyundai BMS software was faulty, as it misapplied LG's recommendations for fast charging.^[^ ^18^ ^]^ This recall caused a financial loss of 900 million USD. In 2024, Mercedes‐Benz recalled over 17 000 EVs worldwide due to a BMS software issue, which could lead to sudden power loss while driving, increasing the risk of crashes.^[^ ^16^, ^17^ ^]^ Regarding stationary battery energy storage systems (BESSs), the American Electric Power Research Institute (EPRI) has created a database tracking their failures and incidents worldwide.^[^ ^21^ ^]^ In their 2024 report,^[^ ^11^ ^]^ the root causes of failures in some incidents are analysed and presented. According to it, more than 43% of failures are partly attributable to the control unit, including the BMS. Between 2018 and 2023, the global grid‐scale BESS failure rate per deployed GWh has dropped by 97%.

Given its important role, the BMS must meet strict requirements in terms of functionality, safety, robustness, and reliability. Requirements are usually formulated in standards. These are not legally binding unless integrated into regulations or stakeholder agreements. Several standards exist that address requirements for BMS features. However, only certain features are covered. This fragmented approach increases the risks of inconsistencies, interoperability issues, and potential safety hazards in real‐world use. For example, in the case of battery fires involving an auxiliary power unit of Boeing 787 Dreamliners,^[^ ^22^ ^]^ investigations showed that the BMS failed to monitor battery cell temperature accurately and to mitigate internal short circuits. Although the battery system was certified by the Federal Aviation Administration (FAA), deficiencies and malfunctions went undetected due to regulatory gaps. Therefore, more complete, detailed standards are needed to reduce BMS failure risks and improve battery safety. These standards must also be regularly updated to keep pace with rapid advancements in battery technologies, whose complexity keeps increasing. Furthermore, all BMS features, and not just certain ones, must be covered to avoid inconsistency issues in real‐world implementations. Hence, a clear understanding and overview of the current regulatory landscape is necessary. Due to limited access to standards, this paper provides an overview of current standards and regulations applicable to BMS in Germany, a key player in global battery technology.

**Figure 1:**
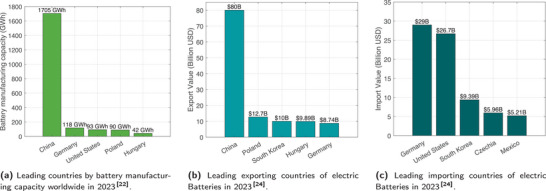
Global leadership of Germany in the battery sector.

According to statistics from 2023 shown in **Figures** **1**a–1c, Germany has the world's second‐largest battery manufacturing capacity (118 GWh) and ranks second in EV production, behind China.^[^ ^23^, ^24^ ^]^ Consequently, it plays a key role in the global battery industry and market. In 2023, Germany was the largest battery importer and the fifth‐largest battery exporter worldwide.^[^ ^25^ ^]^ Given Germany's global position in battery technology and market, this review holds international relevance. Contrary to regulations and law, standards are not freely accessible and typically require purchase. Consequently, this review offers significant value to stakeholders, such as BMS developers and customers, by providing a comprehensive overview of existing standards, thereby saving time and costs. Additionally, institutions responsible for developing BMS‐related standards can leverage the insights from this review to improve current regulations and keep pace with advancements in the battery sector.

The first section of this paper introduces standards for BMS in Germany and presents various classification criteria for BMS requirements. It reviews current requirements. In its second section, it highlights potential improvements and addresses them by making suggestions. The third section of this paper presents some of its limitations and suggests directions for future work.

Due to their high–power and energy density, as well as their low and falling cost,^[^ ^26^ ^]^ the LIBs are currently state‐of‐the‐art for commercial, stationary, portable, and mobile devices. According to recent market reports (2023), LIBs dominate the global battery market, accounting for more than 70% of the sales,^[^ ^27^, ^28^ ^]^ a figure projected to exceed 80% by 2030. Therefore, LIBs hold the focus of this paper.

## 2 Standards and Regulations for BMS in Germany

Standards and regulations are widely used tools addressing requirements for a system/device to improve its quality, safety, uniformity, and interoperability on the market. According to the European Norm *EN 45020*,^[^ ^29^ ^]^ which is also valid in Germany, a standard is a document developed through a consensus‐building process and endorsed by a recognized institution. It establishes rules, guidelines, or specific characteristics intended for widespread and repetitive use in various activities or their outcomes, with the primary objective of promoting an optimal level of organization within a particular context. However, standards can also be endorsed by non‐recognized organizations. For instance, companies have internal standards, called company‐specific standards, for their products and processes (e.g., Tesla with their Etherloop^[^ ^30^ ^]^). Consequently, standards can be categorized into two main categories:

- De jure (mandatory) standards: These standards are issued by recognized organizations and officially mandated by governments to ensure high quality in terms of performance, safety, ethics, and environmental protection. As a result, they are legally binding.
- Voluntary standards: These are not legally binding, but are usually widely adopted because of their benefits in practice.

Voluntary standards involve company‐internal standards, which are unfortunately not widely used and not openly accessible. Hence, the review proposed in this paper only considers voluntary and mandatory standards endorsed by recognized organizations and regulations valid in Germany. Since BMSs are electric/electronic devices, important recognized German institutes responsible for developing, adopting and publishing standards that can be related to BMS are listed below:

- Deutsches Institut für Normung e.V. (DIN): The German Institute for Standardization is Germany's most influential standardization institute. It endorses the development and publication of national standards across a wide range of industries and sectors. DIN also represents German interests in international and European standardization organizations.
- Deutsches Elektrotechnisches Komitee (DKE): The German Commission for Electric, Electronic, and Information Technologies is responsible for standardization in the field of electrical engineering and related technologies.
- Verein Deutscher Ingenieure (VDI): The Association of German Engineers is involved in developing technical guidelines and standards.
- Verband der Automobilindustrie (VDA): The German Association of the Automotive Industry, VDA, is involved in developing standards and guidelines for the automotive industry.
- Verband der Elektrotechnik, Elektronik, und Informationstechnik (VDE): The German Association for Electrical, Electronic & Information Technologies. It is one of the largest technical and scientific associations in Europe. One of its tasks consists of the development of standards and safety regulations in the fields of electrical engineering, electronics, and information technology.

Additionally, Germany is a member of the European Union and other international organizations. Consequently, German standards (for BMS) must comply with European and international standards endorsed by recognized institutions, including:

- European Committee for Electrotechnical Standardization (CENELEC)
- International Organization for Standardization (ISO)
- International Electrotechnical Commission (IEC)
- United Nations (UN)

Based on this, a landscape for standards addressing requirements for BMS in Germany has been created and will be presented in Section 2.1. However, it is important to note that there are numerous standards and regulations whose requirements are directly or indirectly related to a BMS. Not all of these can be comprehensively reviewed in this paper. Consequently, this study will focus on the most significant ones, identified through a literature review and best user practices.

### 2.1 Requirements for BMS in Germany

Depending on the application, there are standards addressing requirements and guidelines for BMSs. A general definition for BMSs is outlined in a current regulation of the European Parliament.^[^ ^31^ ^]^ According to it, the BMS is an electronic component that monitors and controls the electrical and thermal functions of a battery for its safety, performance, and durability (i.e., to increase the battery lifetime). Furthermore, it manages and stores data related to battery ageing and expected lifetime parameters, and communicates with the vehicle, light transport vehicle or device in which the battery is installed, or with a public or private charging infrastructure. This definition highlights the high level of responsibilities linked to a BMS. Similar definitions are provided in standards such as *DIN EN 62620*, *DIN EN 62619*, *DIN EN IEC 63115* for industrial applications, *DIN EN 50604‐1*, *ISO 12405* for battery electric vehicles (EVs), *DIN EN IEC 63056* for BESSs, and *IEC/TS 61851* for conductive charging stations for EVs.

A BMS comprises two principal components:

- Hardware: Physical components of the BMS. Nowadays, it is exclusively implemented using electric/electronic (E/E) devices on printed circuit boards (PCBs) for various reasons, including safety, long‐term performance, volume, and robustness.
- Software: Algorithms implementing strategies used to operate the battery or battery system.

Both components must meet strict requirements to ensure the safe and optimal operation of the battery system. These requirements depend on several factors, including the application, as it defines operating scenarios, environmental conditions, disturbances, and electrical noise, and the battery chemistry (e.g., lithium iron phosphate (LFP), nickel manganese cobalt (NMC), nickel cobalt aluminium oxide (NCA), etc.). Different battery chemistries exhibit singular electrical behaviors, characteristics, and ageing patterns, all of which the BMS must take into consideration to enhance battery performance. For instance, a deep understanding of the battery chemistry (cathode and anode) is essential for precise state of charge (SoC) estimation. In NMC batteries with graphite anodes (NMC/graphite), the open circuit voltage (OCV) is commonly used as a SoC indicator at rest, whereas in LFP/graphite batteries, this is more challenging due to their flatter OCV–SoC curve.^[^ ^32^, ^33^ ^]^

For a comprehensive overview, BMS requirements have been categorised into two main groups:

- Functional requirements: They describe the functions that a BMS must perform to guarantee the safety of a battery or battery system. They may also be extended to encompass performance aspects. The performance of a BMS can be defined differently depending on the application and its objectives. In this paper, it refers to how effectively a BMS performs its tasks in terms of accuracy, speed, and precision.
- Non‐functional requirements: They refer to criteria that define the overall qualities or attributes of the BMS and its features in terms of reliability, efficiency, and robustness. These requirements influence both the BMS hardware and software, and therefore play an important role in the development and implementation of BMSs.^[^ ^34^ ^]^

These requirements can further be divided into two subcategories:

- Qualitative requirements: Non‐numeric, non‐measurable characteristics and attributes of a BMS feature.
- Quantitative requirements: Are expressed in numerical terms and are measurable. These requirements provide specific, quantifiable criteria for assessing the performance of a BMS feature.

The performance criteria for a BMS are defined as follows:

- Accuracy: It is a metric indicating how close a measurement, estimation, or prediction is to the real value.
- Speed: How fast the BMS performs a task.
- Precision: It is the degree to which repeated measurements or estimations under unchanged conditions show the same results.

**Figure 2:**
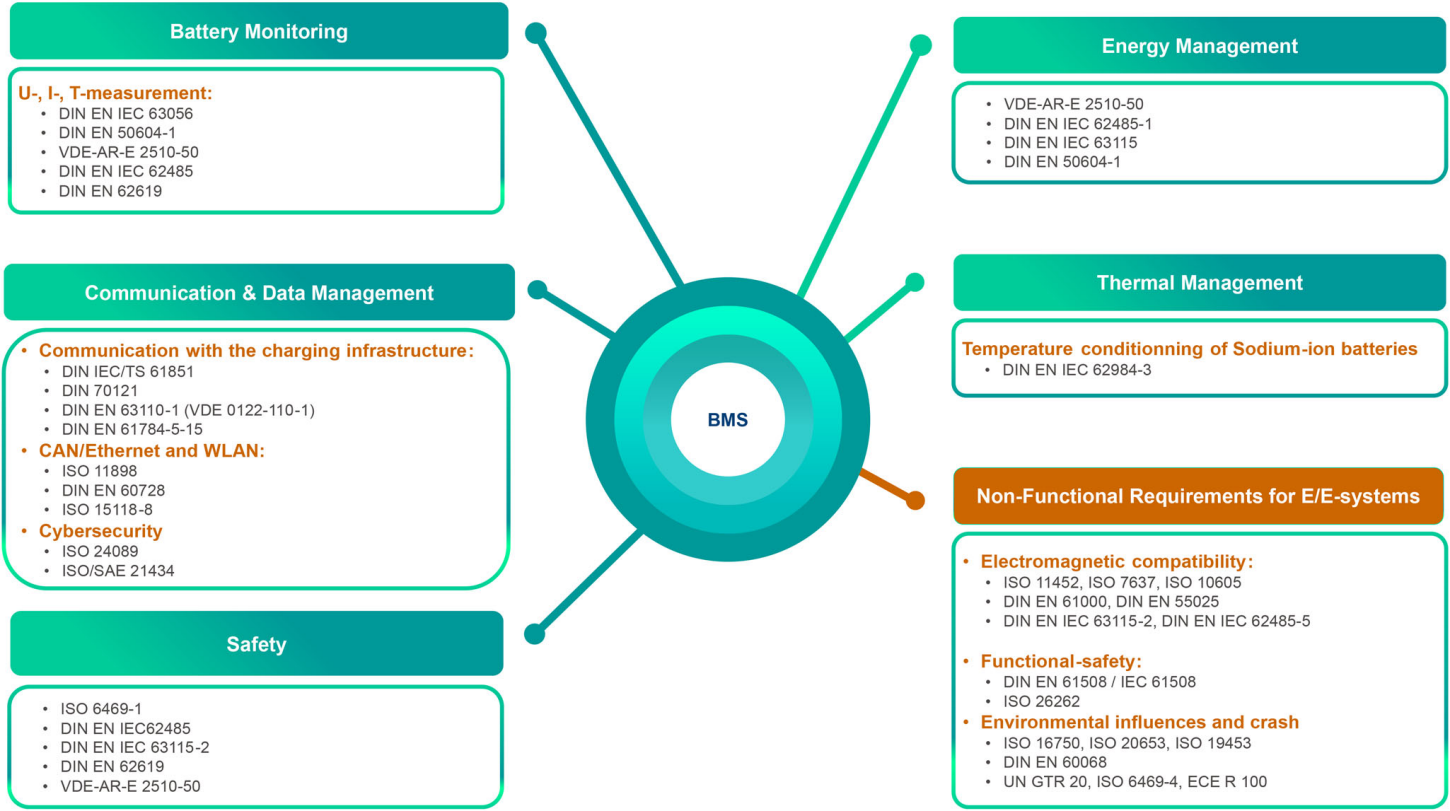
Standards valid in Germany addressing requirements for BMSs.

**Figure** **2** shows an overview of standards valid in Germany, which can be related to BMSs. Its content will be reviewed in detail in the following subsections.

#### 2.1.1 Functional Requirements

Functional requirements refer to those addressed to the core features that a BMS must exhibit to ensure the safety of batteries. These requirements are frequently derived from standards relating to the functional safety of battery systems, which indirectly specify the necessary characteristics of the BMS. This section provides an overview of current standards in Germany that address functional requirements for BMS (see Figure 2).

Typical core features of a BMS, essential for safety in major applications such as EVs and BESSs, are listed below. These are based on valid standards as well as on best practice examples mentioned in Refs. [35, 36, 37, 38, 39, 40]:

- Battery monitoring: It performs measurements and monitors the battery's condition, enabling the BMS to react accordingly and prevent or mitigate potential detrimental circumstances that may deteriorate the battery or pose safety hazards.
- Safety: It prevents safety hazards and degradations by identifying and diagnosing any faults, errors or potentially hazardous conditions that may occur within the battery system and provides appropriate responses by implementing safety protocols.
- Energy management: It regulates the charging and discharging processes of the battery, ensuring it remains within its SOA to prevent safety issues and battery degradation. Therefore, it works closely with the feature “Safety.” Optionally, it employs strategies to optimise both the battery's short‐term (maximizing efficiency and power) and long‐term performance (extending battery lifetime). These strategies include cell balancing that prevents any significant cell‐to‐cell imbalances.
- Communication and data management: It enables data exchange within the BMS, as well as with external devices. Furthermore, it collects, organizes and stores data of the battery system by ensuring its security.
- Thermal management: It controls the battery temperature to guarantee it operates within its safe and optimal temperature windows. This involves heating, cooling, and thermal balancing, which prevent excessive temperature gradients and maintain uniform temperature across the battery.

**Figure 3:**
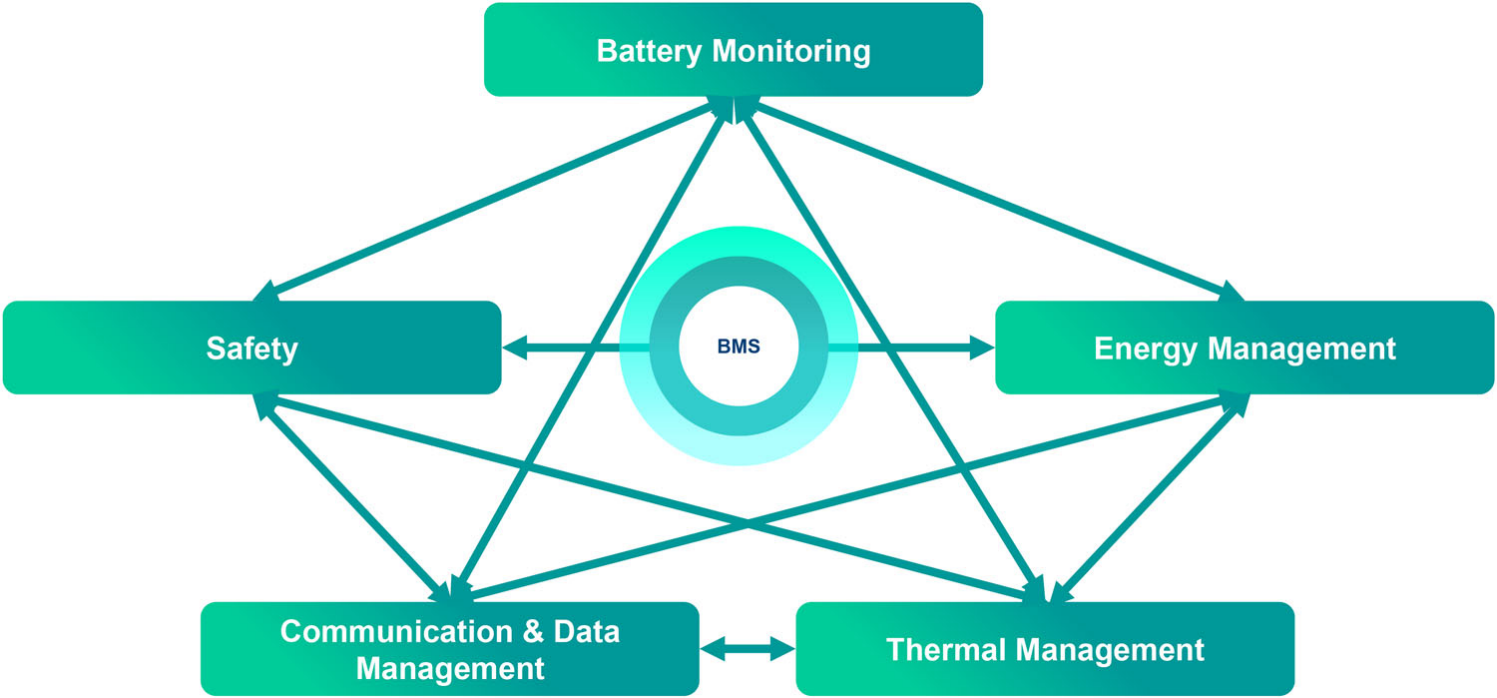
Interdependency between functional features of a BMS.

These features are interdependent, with the output of one serving as input for another. For example, the feature “Safety” relies on the data provided by the features “Battery Monitoring” and “Communication and Data Management.” Furthermore, it requires access to the feature “Energy Management” to control the current flow through the battery. **Figure** **3** illustrates this interdependency of BMS features.

##### Battery Monitoring

It monitors the state of the battery, enabling the BMS to respond appropriately, either by preventing the battery from exceeding its SOA or by mitigating risks as operating limits are approached. The SOA defines the boundaries for the permissible battery's operating parameters and conditions. According to *DIN EN IEC 62619* and established literature,^[^ ^37^, ^41^ ^]^ the SOA for a LIB should be specified by the battery manufacturer. Key parameters defining the SOA include cell and battery voltages, temperature, and charge and discharge current limits. In addition, this function is responsible for monitoring various battery state parameters, such as state of X (SoX) parameters, internal resistance (*R*_*int*_), and electrical insulation resistance. This section reviews the relevant standards and regulations addressing requirements for this feature.

Requirements for battery monitoring vary with applications. Some examples are listed below:

- *VDE‐AR‐E 2510‐50*: For BESSs, mandates the BMS to monitor individual cell or cell block voltages, the overall battery system current, and temperatures at the cell level.
- *DIN EN 50604‐1*: For EVs, requires the monitoring of all battery cell voltages.
- *DIN EN IEC 62485‐6*: Applicable to traction batteries, mandates, in addition to voltage and temperature monitoring at the cell level and current monitoring at the pack level, also current monitoring at the cell level to prevent any overcurrent.

In addition, *VDE‐AR‐E 2510‐50* specifies procedures for validating the BMS's measurement accuracy. It specifies a comparison between the BMS measurements (voltage, internal temperature, and current) and those of a calibrated test bench. Therefore, the values of the BMS and the resulting accuracy are documented. It also addresses a qualitative requirement for the BMS's accuracy: the BMS‐measurement accuracy must be sufficient to ensure that any SOA violation is detected at all times.

In certain applications, the BMS may be tasked to monitor the electrical insulation of the system. Operational requirements for electrical devices and, by extension, for BMS implementations are specified in *DIN VDE 0105‐100* and *DIN VDE 0100‐600* (for pre‐commissioning verification). These standards define specific threshold values for insulation resistance based on system type (AC/DC) and architecture. For instance, a minimum insulation resistance of 1 MΩ is required by *DIN VDE 0100‐600* for electrical systems with a nominal voltage up to 500 V.

##### Thermal Management

It plays a critical role in ensuring the performance, reliability, and safety of battery systems, particularly those with high‐power demands, such as EVs, and systems using high‐temperature batteries. Improper thermal regulation can lead to accelerated degradation, reduced efficiency, or even hazardous conditions. This section provides an overview of standards and regulations related to the thermal management of batteries.

*DIN EN IEC 62984* outlines the requirements for high‐temperature batteries, defined as batteries whose minimum internal cell operating temperature exceeds 100 °C. These batteries require precise thermal control due to their elevated operating conditions. Specifically, *DIN EN IEC 62984‐3*, which applies to high‐temperature sodium‐ion batteries (SiBs), sets detailed requirements for BMS functionality. According to it, the BMS must monitor and regulate the internal battery temperature automatically and independently, without any user intervention. This is essential to prevent damage caused by human error or oversight. To this end, it must automatically manage heating and cooling processes according to the prescribed temperature profile. Additionally, it restricts user access to prevent any modifications of this predefined profile to eliminate the risk of unintended changes. These measures ensure that the battery remains within its safe thermal operating window under all conditions, thereby contributing to the overall safety and longevity of the system.

##### Safety and Energy Management

To ensure the battery remains within its SOA, the features “Safety” and “Energy Management” work closely together and with other features to control the current flow through the battery and to provide an adequate BMS response in case of an error. Numerous standards address requirements related to these features. They are reviewed in this section.

*DIN EN IEC 62485*, *DIN EN IEC 63115*, *DIN EN 50604‐1*, and *VDE‐AR‐E 2510‐50* require the BMS to control the battery current during charging and discharging, and to bring the battery into a safe state if its SOA is violated. Typically, the BMS fulfils this function by interrupting the battery current via a contactor. For this reason, most standards explicitly require the BMS to shut down the current when SOA‐threshold values are exceeded. For example, *DIN EN IEC 63115‐2* and *DIN EN IEC 62619*, applicable to industrial batteries, mandate that the BMS must interrupt the current if cell temperature or current surpasses defined safety limits. *DIN EN IEC 62619* further specifies that the BMS must interrupt the current before or as soon as the cell voltage reaches its upper safety threshold, as the voltage is considered a more critical indicator than current or temperature. In line with this, *ISO 6469‐1*, *VDE‐AR‐E 2510‐50*, and *DIN EN IEC 62485‐5*, which apply to batteries used in BESSs, also require current interruption in the event of overcharging, deep discharge, or overheating. However, an exception is noted in *DIN EN IEC 62485‐6* for LIBs used in EVs. It allows deep discharge when necessary to fulfil critical operational needs, such as enabling the vehicle to exit a safety‐critical situation. Therefore, immediate shutdown of the battery is not always required. In certain cases of SOA violation, particularly those involving current or temperature, alternative responses such as increasing the thermal management system's cooling power or reducing current flow may suffice. According to *DIN EN IEC 62485‐6*, the BMS must either limit or interrupt the charging or discharging current when the battery operates outside its permissible temperature range.

The current interruption function is a fundamental safety mechanism and must comply with the appropriate Safety Integrity Level (SIL) as defined in *IEC 61508*. In automotive applications, the equivalent classification, Automotive Safety Integrity Level (ASIL), is specified in *ISO 26262*. *DIN EN IEC 62485‐5* requires the current interruption to be redundantly implemented for reliability reasons. In systems where the battery is connected to a charger, the communication link between the battery and charger serves a critical safety role. Accordingly, the BMS must also interrupt the battery current in the event of a communication failure.

For high‐temperature batteries, the standards of *DIN EN IEC 62984* additionally require the BMS to restrict user access to prevent any modification of the predefined temperature profile and to maintain thermal regulation when a power supply failure occurs. Furthermore, the BMS must prevent charging and discharging outside the specified internal temperature range and inhibit such operations during heating or cooling phases, or when the battery has not yet reached its designated operating temperature.

##### Communication and Data Management

Batteries are used in systems as power sources for other system components. For safety and efficiency reasons, they and thus their BMS should be able to communicate with surrounding systems and devices. In many applications, such as EVs and BESSs, communication between the battery (via the BMS) and external devices (e.g., chargers, power electronics, or other ECUs: electronic control units) is safety‐critical. This is particularly true for EVs, where reliable and real‐time communication between the BMS and various ECUs is essential for ensuring safe and optimal operation of the overall system. To meet these communication demands, the BMS must provide appropriate and application‐specific communication interfaces and protocols. For instance, the Controller Area Network (CAN) is the standard in EVs, whereas industrial and stationary applications typically use CAN or Modbus TCP. In this context, standards such as *ISO 11898* (for automotive CAN communication) and *DIN EN 61784‐5‐15* (for industrial communication networks) may be indirectly applied to BMS‐related communication requirements.

Ethernet‐CAN is gaining popularity among OEMs due to its higher bandwidth and reduced wiring complexity compared to traditional CAN. For example, Tesla and BMW^[^ ^42^ ^]^ have already implemented Ethernet‐CAN in certain vehicle models (the Etherloop in the Tesla Cybertruck^[^ ^30^ ^]^). As a result, globally adopted standards such as *IEEE 802.3‐2018* may be relevant for BMS communication requirements.

Another critical communication path for the BMS is with the EV charger. Most chargers for EVs (including PHEV: Plug‐in Hybrid Electric Vehicles) operate using direct current (DC). According to *DIN 70121*, the BMS must be capable of real‐time communication with the external charger to ensure safe and optimized charging. The standard specifies requirements for both the communication protocol and architecture. Similarly, *DIN EN 61851‐24*, related to DC charging stations for EVs, addresses the quality and reliability of digital communication between the BMS and the charger. It defines the necessary exchange of critical parameters such as maximum voltage, current, SoC, SoH, and error messages, as well as the corresponding response protocols.

Furthermore, *DIN EN 61851‐3‐4* introduces the requirement for a compatibility check before initiating the charging. The BMS must provide key identification data, such as device type, serial number, manufacturer, and operating limits, to verify compatibility with the charger. Charging may only proceed once this compatibility has been successfully established.

While conductive charging is currently the dominant method for EVs, inductive (wireless) charging is expected to become increasingly used. In this context, *DIN EN IEC 61980‐2* and *IEC 61980* outline requirements for wireless communication during charging. For example, the communication with the charger should be carried out via a Wireless Local Area Network (WLAN) and in accordance with *ISO 15118‐8*.

Emerging technologies such as wireless BMS (wBMS) and cloud‐based BMS solutions are gaining attention. These systems often rely on wireless communication standards such as those for WLAN. Consequently, recognized standards like *DIN EN 60728‐1‐1* and *IEEE 802.11* may indirectly have relevance.

Advanced BMS concepts (wBMS, cloud BMS, IoT: internet of things) also introduce significant cybersecurity challenges. As a result, compliance with cybersecurity standards is essential. Relevant automotive‐specific cybersecurity standards include *ISO/SAE 21434* and *ISO 24089*. More broadly, *DIN EN IEC 62351* provides general cybersecurity requirements for energy management systems, while *DIN EN ISO/IEC 27001* offers a framework for information security management. Additionally, the REGULATION OF THE EUROPEAN PARLIAMENT AND OF THE COUNCIL ON HORIZONTAL CYBERSECURITY REQUIREMENTS, adopted in October 2024, will further shape regulatory expectations in this area.

#### 2.1.2 Non‐Functional Requirements

Non‐functional requirements usually describe BMSs relatively of their efficiency, reliability, and robustness. These requirements strongly influence both the hardware and software aspects of BMS design, including materials, architecture, and system integration. This section reviews key non‐functional requirements and the corresponding standards that can be applied to BMSs.

Due to their large application fields, batteries and thus BMSs are exposed to different conditions and disturbances and should be robust against these. The robustness of the BMS refers to its ability to maintain a constant performance within unexpected conditions. An important feature of E/E devices and thus BMSs is their robustness against electromagnetic interference. Due to their wide spectrum of applications, batteries can be subject to various electromagnetic environments, including home, industrial, and commercial areas. Therefore, the requirements outlined in international standards of the *IEC 61000/61326* series are helpful guides for electromagnetic compatibility (EMC) requirements for BMS. They are related to electromagnetic emission (EME), immunity (EMI), and electrostatic discharge (ESD). These standards also include methods for testing and validating compliance.

Beyond the general electromagnetic environment, EMC requirements are often application‐specific. For example, *ISO 11452* defines test procedures for components in road vehicles exposed to electrical disturbances. For EVs using wireless charging, EMI and EME requirements are specified in *DIN EN IEC 61980‐1*. International EMC standards are often transposed into national equivalents, such as the *DIN EN IEC 61326* series, which applies to measurement, control, and laboratory equipment, making it applicable to BMSs as well.

Additionally, BMSs must not interfere with the operation of other E/E devices in a system. *DIN EN IEC 62619* indirectly addresses this by requiring EMC compatibility testing for the entire battery, including the BMS, in relation to other connected components before it can be incorporated in a system. Similarly, *DIN EN IEC 63115‐2* mandates that a battery system, including its BMS, must meet the EMC requirements relevant to the specific application or as agreed upon with the end product manufacturer.

Functional safety is another critical area influencing non‐functional requirements. International standards such as *IEC 61508*, *ISO 26262* (for E/E systems in series‐production road vehicles), and *ISO 6469* (specific to electric vehicles) define the required safety integrity levels (SIL/ASIL) and impact both the hardware and software design of the BMS. For example, *ISO 6469‐3* specifies that components exposed to class B voltages or higher must include high‐voltage markings. Since BMSs are often connected to batteries operating above 60 V (DC) or 30 V (AC), this requirement directly applies. Furthermore, *DIN EN 61508‐3* outlines specific development practices for the software of programmable electronics to ensure functional safety compliance.

In addition to electrical robustness, BMSs must also withstand environmental constraints, such as extreme temperatures, mechanical shock, pressure, humidity, water, and dust. Given the wide geographic distribution of batteries, the BMSs must operate reliably in a broad temperature window. For instance, at temperatures exceeding 45 °C in Saudi Arabia or below –30 °C in Siberia.^[^ ^43^ ^]^ In EVs, the BMS is exposed to frequent mechanical stresses due to vehicle acceleration and must also be able to function properly during and after a crash event. Standards such as *UN GTR 20*, *ECE R 100*, and *ISO 6469‐4* specify safety requirements for EV batteries in such scenarios. These standards ensure that users are not exposed to hazardous voltages during or after a collision. Compliance can be demonstrated by meeting at least one of the following criteria:

- Absence of high voltages: Voltage levels remain within class A (*U* < 30 *V* (AC) or 60 *V* (DC)).
- A high isolation resistance between the high‐voltage bus and the vehicle chassis.
- Physical barriers covering all exposed electrified components.

EVs are generally equipped with contactors (relays) designed to disconnect the battery automatically in case of a crash or fault. These contactors are often controlled by the BMS. As outlined in Refs. [44, 45], the crash test must validate the correct function of the automatic disconnection mechanism, which should remain operational after the crash.

Like all electronic equipment marketed in the European Union, BMSs must comply with environmental directives, particularly regarding hazardous substances. The Restriction of Hazardous Substances (RoHS) directive,^[^ ^46^ ^]^ transposed into German law via the ElektroStoffV,^[^ ^47^ ^]^ sets limits on the concentration of certain materials and mandates requirements for sustainability and recyclability.

**Table** **1** provides an overview of other key standards addressing non‐functional requirements for E/E systems, and by extension, BMSs.

**Table 1:** Non‐functional requirements applicable to BMSs.

Fields	Functionality/Aspects	Standards/regulations
Electromagnetic compatibility	Electromagnetic interference emission	*DIN EN IEC 62485‐5*, *DIN EN 61000‐6‐3*, *DIN EN 55025*, *DIN EN IEC 61980‐1*
	Electromagnetic immunity	*DIN EN 61000, DIN EN IEC 62485‐5, ISO 11452, ISO 7637*
	ESD	*ISO 10605, DIN EN 61000*
Functional safety	Hardware	*DIN EN 61508‐(1,2), ISO 26262‐(2,5), ISO 6469‐1, ISO 6469‐3*
	Software	*DIN EN 61508‐3, ISO 26262‐6*
Reliability against external influences	Mechanical shocks (crash/crush), temperature, pressure, humidity, water, and dust	*ISO 16750, DIN EN 60068, ISO 20653, ISO 19453, UN GTR 20, UN ECE R 100, ISO 6469‐4*
Environmental impact and sustainability	Concentration of hazardous substances and recycling	EU‐RoHS, ElektroStoffV

## 3 Improvement Potentials

A review of BMS standards in Germany revealed numerous standards addressing functional and non‐functional requirements for BMSs, either directly or indirectly. However, these are mostly qualitative and therefore incomplete. The resulting gap can lead to inconsistencies in practical implementations, and thus to interoperability problems between different BMSs. Furthermore, it makes it challenging to benchmark BMSs and hinders the implementation of safety enhancements. In this section, propositions for improving the current state of BMS requirements are presented. A key parameter guiding the proposals and analyses is the BMS performance.

### 3.1 Battery Monitoring

Monitored battery parameters, such as voltage, current, temperature, and the various state indicators, are utilized in energy management, battery control, and safety algorithms. A review of currently applicable standards in Germany has shown that these requirements are largely qualitative and consequently incomplete. Key state parameters, including the state of X (SoX) parameters and internal battery resistance *R*_*int*_ are not yet fully addressed. **Table** **2** provides an overview of the lacks and potential points for improvement in the current requirements for battery monitoring. In particular, it underscores the absence of defined performance requirements for battery monitoring functionalities. One important functionality closely linked to battery monitoring is battery state prediction. Typically integrated into BMS algorithms, state prediction is used to enhance or optimize battery safety, performance, and utilization. For this reason, it is also identified in Table 2 as a functionality with considerable potential for future standardization.

**Table 2:** Lacks and improvement potentials in current requirements for the battery monitoring.

Functions	Aspects	Lacks/Improvement potentials
Measurements	Functionality	Consistencies in standards regarding which parameters to measure and at what level
	Performance	Quantitative requirements for the measurement quality or performance
Estimations	Functionality	Consistent definitions of state parameters across standards
	Performance	Quantitative requirements for the BMS's state estimation in terms of performance
Predictions	Functionality	Define which state parameters should be predicted
	Performance	Quantitative requirements for the BMS's state prediction in terms of performance

To address the points mentioned in Table 2, this section presents suggestions for requirements for future standards. Additionally, it highlights the necessity of establishing standardized testing and validation methods to support the implementation and verification of the proposed requirements.

#### 3.1.1 Measurements

The review in section 2.1.1 revealed that no standard addresses quantitative requirements for the performance of the battery monitoring. Therefore, propositions are made in this section and mostly rely on the state‐of‐the‐art and best practice examples review in the literature.^[^ ^33^, ^36^ ^]^

BMSs typically only measures the voltage, temperature, and current of a battery. Voltage measurement is commonly performed by analog‐front‐end (AFE) integrated circuits.^[^ ^48^, ^49^, ^50^, ^51^, ^52^, ^53^, ^54^ ^]^ Their measurement accuracy usually ranges between ±0,8 mV and ±2 mV. To determine the most suitable AFE for a specific application, several factors are considered, including the desired safety level, cost constraints, and the battery chemistry. Future standards could consider a maximum measurement error of ±1 mV maximum absolute error (MAE) for battery cell voltages. For safety reasons, the voltage monitoring is usually performed at the cell and module levels. This should be mandated by future standards. In applications with high‐energy and power demands (e.g., EVs and BESSs), battery packs are used with system voltages typically exceeding 400 V and reaching up to 800 V for current EVs.^[^ ^55^, ^56^ ^]^ Therefore, it is advisable to also specify a measurement accuracy at the pack level. Given the high voltage values, it may be more appropriate to formulate the measurement accuracy as a relative accuracy to the pack voltage. According to, ^[^ ^33^, ^36^ ^]^ the relative measurement accuracy is traditionally below 0,1% over the full voltage range. This value could serve as a guideline for the maximum permissible measurement accuracy in future standards for battery pack voltage.

The current measurement is an essential feature of a BMS. For cost and technical feasibility reasons, the current is almost exclusively monitored at the pack level. This practice should be mandated by future standards. In best‐practice applications,^[^ ^33^, ^36^ ^]^ the measurement accuracy is typically specified for the full battery scale and ranges between ± 0,5% and ±1%. Given the critical role of current measurement in estimating battery state parameters, future standards could consider ±1% as the upper limit for permissible measurement accuracy. Since the current signal is commonly used in incremental SoC estimation algorithms (e.g., Coulomb counting), where it is integrated over time, specifying a maximal root‐mean‐square error (RMSE) of 1% in addition to absolute accuracy or error (MAE) could help limit the long‐term impact of measurement errors on SoC estimation accuracy.

The temperature measurement of the BMS depends significantly on the temperature sensor used. For typical civil and industrial applications, resistive thermal sensors (NTCs: negative temperature couplers or PTCs: positive temperature couplers) are used. Their measurement accuracy usually ranges between ±1 K and ±2 K, which is generally sufficient to ensure a reliable thermal management and battery safety. In safety‐critical or high‐performance applications, such as aerospace, medical devices, or automotive systems with high functional safety requirements, higher accuracy could be required. In such cases, sensors with accuracies up to ±0,5 K are commonly employed to enable more precise thermal control and early detection of abnormal thermal behaviors. Future standards could distinguish between general and safety‐critical applications, recommending a maximum temperature measurement accuracy of ±2 K for standard use and ±0,5 K for critical systems. In practice, every battery‐surface temperature is usually not monitored, as it would be linked with high costs and technical complexity. Instead, temperature sensors are placed at specific positions, where hotspots are more likely to occur. Considering its importance for battery safety, a requirement should be formulated, mandating the BMS to monitor the temperature in a way that any temperatures beyond its SOA are detected.

Besides measurement accuracy, another critical characteristic of battery monitoring is the measurement frequency or the refreshing rate. It indicates how often (Hz) the BMS provides new values to its internal components, algorithms, and external devices. Therefore, it sets boundaries for the speed of algorithms. It can be differentiated between:

- Internal refreshing rate/monitoring frequency: It indicates how frequently the BMS internally updates new values for the monitored parameters.
- External refreshing rate/monitoring frequency: It defines how often the BMS transmits new values of monitored parameters to external components and devices. It is usually less than the internal refreshing rate.

Despite its key role in ensuring battery system safety and performance, current standards do not specify requirements for the measurement frequency. A high internal monitoring frequency enables near real‐time tracking of system conditions, thereby increasing the system's responsiveness and safety. It allows the BMS to detect anomalies promptly and improves the reliability of state estimation algorithms. Gu et al.^[^ ^57^ ^]^ experimentally demonstrated that increasing the sampling frequency of BMS measurements enhances the accuracy of battery models and the precision of SoC estimations. Therefore, defining minimum requirements for battery monitoring frequencies would significantly improve both BMS reliability and overall battery safety and performance. Thereby, care must be taken, as too high monitoring frequencies can lead to data overflow and BMS failures. Furthermore, monitoring frequencies should be tailored to the dynamics and priority of the specific parameters. For example, voltage and current measurements typically require higher internal monitoring frequencies compared to temperature due to their more dynamic nature, especially in applications such as EVs and HEVs. The monitoring frequencies typically range from 100 Hz (10 ms) to 1 kHz (1 ms), with some applications/scenarios demanding up to 10 kHz for real‐time monitoring. In contrast, the temperature changes more slowly and is generally monitored at frequencies between 1 Hz (1 s) and 10 Hz (0,1 s). Regarding the external monitoring frequency, it is usually lower than the internal one and strongly depends on the application. It must be sufficiently high to ensure reliable monitoring, but not too high to avoid data overload or memory management issues. Therefore, future standards could define the following minimum internal monitoring frequencies:

- Internal frequency monitoring:
  – Voltage and current: At least 100 Hz
  – Temperature: At least 1 Hz for high‐power applications and 0,1 Hz for high‐energy applications
- External frequency monitoring: Is application‐specific and is usually less than the internal monitoring frequency.

A further improvement potential revealed by the review is the insulation monitoring. In applications involving many systems, such as EVs, the insulation monitoring typically falls into the responsibility of higher controllers. However, it may fall in certain applications under the responsibility of the BMS. Therefore, it should be able to measure the insulation resistance of the whole system. The required insulation resistance varies based on the system type (AC/DC) and its architecture. It is typically high (10 kΩ to several MΩ). Given this high value, specifying a relative accuracy of 1% may be more appropriate than an absolute one.

Propositions for measurement performance can be summarized as follows:

- Unit:
  – Voltage: mV
  – Current: mA
  – Temperature: K
  – Insulation‐resistance: kΩ
- Accuracy:
  – Voltage: ±1 mV MAE at cell level and 0,1% at module or pack level
  – Current: ±1% MAE and less than 1% RMSE at module or pack level
  – Temperature: Less than ±2 K MAE
  – Insulation‐resistance: Less than ±1%
- Refreshing rate/measurement frequency (speed):
  – Internal refreshing rate:
    * Voltage and current: At least 100 Hz (10 ms) for high‐energy applications and 1 kHz for high‐power applications or scenarios
    * Temperature: At least 1 Hz (1 s)
    * Insulation‐resistance: At least 1 Hz (1 s)
  – External refreshing rate:
    * Voltage and current: Is application‐specific. As orientation, at least 10 Hz (100 ms)
    * Temperature: At least between 0,1–1 Hz (1–10 s)
    * Insulation‐resistance: At least 1 Hz (1 s)

Upcoming standards should define test and validation procedures that also include specifications for measurement precision, for example, based on the standard deviation σ.

#### 3.1.2 Estimations

The battery state estimation is a crucial BMS feature, as it is traditionally used for safety‐ and performance‐relevant tasks. There are numerous state parameters, including SoX parameters (SoC, SoH, state of power (SoP), state of energy (SoE), state of safety (SoS), state of functionality (SoF)) and *R*_*int*_. Due to their critical role in battery operational safety, SoC and SoH are the most widely utilised in practice. Accordingly, this section focuses on these two parameters.

The SoC and SoH cannot be directly measured from the battery. They are always estimated using the BMS measurements and algorithms. Considering their importance, SoC‐ and SoH‐estimation algorithms should meet several requirements (functionality and performance) before being integrated into real‐life applications. However, this is challenging due to inconsistent definitions and implementations in real‐life practices. These aspects are detailedly developed in the following subsections.

State estimation algorithms are numerous, including model‐based, data‐driven, and hybrid methods. Recent literature reviews^[^ ^58^, ^59^, ^60^ ^]^ reveal a trend of using data‐driven (machine learning) methods, which usually enhance the performance of traditional methods (model‐based). However, the proposed requirements presented in the following subsection purposely do not require their utilisation or any other methods. The authors view the role of standards in this instance as defining performance targets while allowing developers the flexibility to choose the most suitable approach to achieve those targets.

The performance of state parameters highly relies on the quality of the BMS's measurements.^[^ ^61^ ^]^ Therefore, propositions in this regard are presented in the subsection Measurements.

##### SoC

The SoC is an indicator of the current charge level of a battery, supporting tasks such as estimating driving range in EVs or managing charge/discharge cycles in BESSs. The SoC is typically expressed as a percentage (%), with 100% representing a fully charged battery and 0% a fully discharged one. These SoC limits (full and empty) are generally defined by voltage and current thresholds. For instance, 0% SoC is typically reached when the battery voltage falls below the minimal permissible battery voltage *V*_*min*_. The thresholds for 100% SoC are more complex, as special charging protocols are often required to charge a battery fully. These thresholds are defined based on the SOA. However, users typically apply more conservative limits for safety or battery longevity purposes.

Depending on the perspective or application sector of the battery, the definition of SoC can vary. Accordingly, as noted in^[^ ^62^ ^]^ a distinction can be made between two main interpretations:

- True/theoretical SoC (t‐SoC): It is used in scientific contexts and reflects the theoretical battery SoC in its thermodynamic equilibrium state, where chemical, electrical, and mechanical conditions are fully stabilised.
- Engineering SoC (e‐SoC): It is a more practical and user‐oriented SoC definition. In contrast to the t‐SoC, its estimation or calculation accounts for constraints arising from real‐world applications.

Due to the constraints required for thermodynamic equilibrium, the t‐SoC can only be measured or estimated under controlled conditions (e.g., temperature and pressure) and with a small load current. A similar approach is described in *DIN EN IEC 62864*. However, these conditions are only achievable in laboratory environments and not in real‐life applications. Therefore, the e‐SoC is preferred for practical use. In this context, a basic definition of the e‐SoC has been proposed in *DIN EN IEC 62660*. It defines the SoC as the current battery cell capacity *Q*_*res*_ expressed as a percentage of its nominal capacity *Q*_*Nom*_. The nominal capacity, *Q*_*Nom*_, refers to the battery cell capacity in its fresh state. It is usually specified by the manufacturer.

(1) $$\begin{array}{cc} SoC & =\frac{Q_{res}}{Q_{Nom}}\cdot 100\;\% \end{array}$$

**Figure 4:**
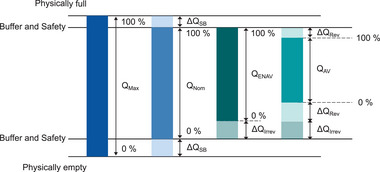
Different definitions of the reference capacity according to [64].

This definition seems ambiguous and incomplete by considering the complexity of battery capacity at the module level and other influences, such as battery degradation, temperature, and load.^[^ ^63^ ^]^ These dependencies make it challenging to formulate a unified SoC definition. This challenge is highlighted in [^64^, ^65^ ^]^]. According to them, the main issue lies in defining the denominator, the reference capacity, for Equation 1. To address this, Sauer et al. have introduced different reference capacities for the SoC definition, which consider the battery ageing state, temperature and load.^[^ ^66^ ^]^ These are qualitatively illustrated in **Figure** **4**. After manufacturing, a battery has a maximum theoretical capacity *Q*_*Max*_. However, for safety and long‐term durability reasons, only a portion of this capacity, called the nominal capacity (*Q*_*Nom*_), is made available for use. Δ*Q*_*SB*_ is the safety/buffer‐capacity reserve. Over time, the battery undergoes ageing/degradation, both from regular use (cyclic ageing) and storage (calendar ageing). This leads to irreversible capacity loss, represented by Δ*Q*_*Irrev*_, and results in a reduced effective capacity *Q*_*ENAV*_. In addition to ageing, reversible factors such as temperature and load current also impact the available capacity. These are represented by Δ*Q*_*Rev*_. For instance, low temperatures or high current loads can temporarily reduce performance. LIBs are particularly sensitive to charging protocols. Special charging methods are required to safely fully charge batteries. Depending on the used protocol, the usable capacity may temporarily increase or decrease, affecting how much energy can be drawn from the battery. These reversible effects are considered by *Q*_*AV*_, which is defined as the available capacity after an operating charge and at the actual temperature. Considering this complexity, an improved SoC definition is proposed in *DIN EN IEC 62984* and presented in Equation 2. It defines the SoC as the ratio (in %) of the current available/usable electrical charge in a battery and the fully charged state. Therefore, this definition indirectly includes stress factors noted above. Similar definitions are proposed in other standards, such as *DIN EN IEC 62928*, and in the literature.^[^ ^60^, ^67^ ^]^

(2) $$SoC=\frac{Q_{res}}{Q_{AV}}\cdot 100\;\%$$

However, there is still a discrepancy across standards and literature. For instance, *DIN EN IEC 62933* for BESSs presents an energy‐based approach for the SoC. According to it, the SoC is a ratio between the available energy and the current energy capacity, usually expressed as a percentage. It includes the voltage in Equation 1, increasing the SoC complexity. **Table** **3** gives an overview of the different existing SoC definitions across some standards and legislations valid in Germany.

**Table 3:** Discrepancies in SoC definitions across various standards.

Reference	Classification	SoC Definition
*DIN EN IEC 62864*, *DIN EN IEC 62928*	Capacity based	Ratio (in %) of the current available/usable electrical charge in a battery and the fully charged state
*DIN EN 61982*, *DIN CLC IEC/TS 61851* (^*^)	Capacity based	Available capacity of a battery, given as a percentage of the nominal capacity
*DIN EN IEC 62660*	Capacity based	Capacity of a cell, expressed as a percentage of the nominal capacity
*DIN EN IEC 62933*, EU‐battery passport^a)^	Energy based	Ratio between the available energy and the current energy capacity

^*^: most used definition in the theoretical analyses according to.^[^ ^65^ ^]^

^a)^ The EU‐battery passport is a digital act containing information about a battery regarding its origin, composition, and performance. It aims to improve transparency, traceability, and sustainability in the battery supply chain, especially for batteries above 2 kWh.

Inconsistencies across standards can increase the risk of divergent implementations in real‐life applications, leading to interoperability issues. Different SoC definitions may also result in inaccurate estimations, which can cause overcharging or deep discharging, potentially leading to thermal runaway, fires, or accelerated battery degradation.^[^ ^34^, ^68^, ^69^, ^70^ ^]^ According to,^[^ ^71^ ^]^ for battery systems used for grid frequency control, inaccurate SoC can lead to financial losses, as it can reduce their operating time and thus their profitability. From the user's perspective, inaccurate SoC readings can lead to confusion and reduced trust in battery‐powered devices. For example, an electric vehicle displaying a full charge based on nominal capacity, while the actual capacity is affected by degradation, temperature, or load, may result in unexpected range limitations. Furthermore, such inconsistencies hinder the benchmarking of SoC estimation methods.^[^ ^65^ ^]^ In the case of SoC estimations based on machine‐learning approaches, which require large datasets for training, inconsistent SoC estimations can lead to inconsistencies in training data, making model training more challenging.^[^ ^72^ ^]^

Given its dominance across the research and engineering fields, future standards should consider a capacity‐based SoC definition. Despite its widespread adoption for theoretical analyses, the definition from *DIN EN 61982* is not suitable for practical usage. Here is an illustrative example. Consider a five‐year‐old EV charged to 100% SoC in a garage at 25 °C during winter. When driven outside into 0 °C weather, its internal resistance, *R*_*int*_, increases. It results in greater voltage drops during discharge, causing the terminal voltage to hit *V*_*min*_ earlier. Although the battery is fully charged, less capacity can be extracted under these conditions. Should the SoC estimation consider *Q*_*Nom*_ or *Q*_*AV*_ as a reference? By considering *Q*_*Nom*_, the SoC displayed on the user interface can never reach 100% due to the irreversible degradation after five years operation and should decrease to reflect this reduced available capacity. However, such adjustments on reversible effects could lead to noticeable SoC fluctuations, potentially confusing users. To prevent this, OEMs often present a stable SoC value, based on *Q*_*AV*_ and instead adjust the estimated driving range to reflect performance limitations. Therefore, future standards should use the definition provided in *DIN EN IEC 62928* or an improved one, such as defining SoC as a ratio in % between the current available, usable battery charge *Q*_*Curr*, *AV*_ and its maximal available charge *Q*_*AV*_ under the same conditions.

In real‐world applications, BMS algorithms for the SoC estimation typically use different SoC definitions, implementation techniques and validations, making it challenging to objectively assess and benchmark their performances. For example, the validation of SoC algorithms traditionally consists of calculating their accuracy compared to a true value under various conditions. However, the accuracies in the literature are calculated using different metrics, including the mean error value over time, the absolute errors (AE), MAE, as well as the RMSE.^[^ ^34^, ^58^, ^59^, ^73^ ^]^ This makes it challenging to standardise the accuracy. An improvement potential for future standards may consist of addressing performance criteria for the BMS estimation algorithms. These could include the following parameters:

- Unit: The percentage (%) is widely used for expressing the SoC and should therefore be uniformly adopted in future requirements.
- Resolution: A value of 0,01% can be appropriate, as it is already specified in standards related to conductive EV charging, such as *DIN CLC IEC/TS 61851*.
- Accuracy: It consists of addressing top limits for the MAE and RMSE, as these are widely adopted metrics for the accuracy.^[^ ^59^, ^73^ ^]^ Furthermore, it should reflect the current state‐of‐the‐art, which lies between ±1% − ±3% MAE and under 3% RMSE, according to the review presented in Refs. [58, 73] and the interviews conducted by Berger et al.^[^ ^34^ ^]^ with different stakeholders (engineers) in the research, and across different industry sectors.
- Refreshing rate (speed):
  – Internal refreshing rate: It should be less than 0,1 Hz (10 s) for high‐power, high‐dynamic applications (EVs and HEVs) and less than 0,05 Hz (20 s) for high‐energy applications such as BESSs.^[^ ^34^ ^]^
  – External refreshing rate: It is usually less than the internal refreshing rate and should be application‐ and parameter‐specific.

Furthermore, future standards should consider standardised validation methods that take the specific application into account. However, this is challenging, as many factors influence the performance of SoC algorithms, such as battery chemistry, temperature, cell‐to‐cell variations (capacity, internal resistance, SoC imbalance), load profiles, and even the BMS hardware. The authors of Ref. [34] have taken an important step in this direction by presenting validation procedures for SoC algorithms that, in addition to accuracy, also evaluate the robustness of the algorithms regarding measurement noises, temperature, initial conditions, battery chemistry, and application‐representative load profiles. Their approach can serve as a useful orientation for future BMS standards. However, their study does not consider precision as a validation criterion. A possible proposition for including precision would be to use the standard deviation, σ, of the SoC estimation over many tries.

##### SoH

The SoH is an important battery state parameter, indicating the health level of a battery. Therefore, it is used to estimate or predict the remaining useful lifetime (RUL) and/or the end‐of‐life (EoL) time for batteries. Battery characteristics evolve with their degree of degradation. Hence, the SoH is typically incorporated into battery safety and control algorithms to enhance the battery performance.

A basic and widely adopted SoH definition considers it as the ratio between the current maximum battery capacity *Q*_*AV*_ and its nominal capacity *Q*_*Nom*_, both measured under nominal conditions.^[^ ^74^, ^75^, ^76^ ^]^ A similar definition is provided in *DIN EN IEC 62840*. However, this definition appears to be incomplete, as battery health should include additional parameters beyond the capacity *Q*, such as:

- *R*_*int*_: *R*_*int*_ tends to increase with ageing, leading to higher heat generation and greater energy losses. For applications with high‐power demands, it is a critical parameter and should be considered in SoH estimation.
- Battery power *P*: It represents the rate at which the battery consumes or delivers energy over time. As *R*_*int*_ increases with ageing, *P* decreases, which can limit the battery's ability to meet high‐power demands, such as those HEVs or BESSs used for grid‐frequency stabilisation.
- Battery efficiency η: It is a metric, indicating how effectively a battery converts the energy it receives (during charging) into usable energy (during discharging). The efficiency η typically declines with ageing, resulting in higher energy losses and reducing the amount of usable charge available during operation.
- Self‐discharge rate: It indicates the amount of charge the battery loses over a specified period and under defined conditions while at rest, i.e., without any external load connected. It tends to increase with battery ageing.

In this regard, some standards and regulations have proposed an improved definition for the SoH. According to the EU regulation for the battery passport,^[^ ^31^ ^]^ the SoH expresses a ratio in % of the current battery performance to its nominal/rated performance. A similar definition is proposed in *DIN EN IEC 62933* for BESSs. However, it mandates that the SoH must also include temporary functional limitations due to malfunctions within the BESS. In this regard, reversible capacity losses Δ*Q*_*Rev*_ can lead to fluctuations in the SoH and to confusion in practice. Furthermore, the valid standard *VDE‐AR‐E 2510‐50* considers only the capacity and *R*_*int*_ as performance indicators. This inconsistency in the SoH definition is summarized in **Table** **4**. The current amendment to the EU battery passport^[^ ^77^ ^]^ considers, in addition to *Q*, and *R*_*int*_ also *P*, η, and even the energy as indicators for the battery performance. As European regulations take precedence over German national ones, future standards should adopt the definition provided there. In addition,^[^ ^77^ ^]^ mandates the BMS to predict the SoH and thus the battery RUL or EoL. In this regard, future standards should provide a unified definition for the EoL. According to this, the EoL of a battery is reached when it cannot fulfil the application's load profile. In this case, the SoH should be marked as 0%, even if the battery still has usable capacity and could be used in less demanding applications. However, the threshold for an EV's EoL is usually defined at 80% SoH. For uniform definitions, future standards should consider the EV's definition, as it reflects that the battery still has usable capacity. A SoH of 0% may confuse the users.

**Table 4:** Discrepancies in SoH definitions.

Reference	Classification	SoH Definition
*DIN EN IEC 62840*	Capacity based	Percentage of remaining maximal battery capacity in comparison to nominal capacity
*VDE‐AR‐E 2510‐50*	Performance based	performance (e.g. in terms of capacity and/or internal resistance) of the aged battery cell compared to the new condition
EU battery passport, *DIN EN IEC 62933*, *DIN EN IEC 62928*	Performance based	performance of the aged battery compared to its nominal/initial state
*DIN VDE V 0510‐200*	Energy based	Ratio of the currently usable energy content at a given time to the originally usable energy content of a battery, expressed as a percentage

Future requirements for SoH estimations can consider the following suggestions:

- Unit and resolution: In percentage % and a resolution of 0,01%, similar to the SoC
- Accuracy: It should reflect the state‐of‐the‐art, which is between 1% – 3% MAE and less than 3% RMSE in accordance to.^[^ ^34^, ^75^ ^]^ An accurate SoH estimation enables the prognostic of the battery's RUL and enhances battery safety, as their SOA can shift with ageing. Additionally, it enables operators and users of battery‐powered devices to perform timely maintenance, helping to avoid costly downtime and unexpected disruptions in battery‐dependent applications.
- Refreshing rate: it should be defined following application‐specific requirements:
  – Internal refreshing rate: As the SoH is not crucial and requires a high computational power, it can be updated less frequently than the SoC. In this regard, its refreshing time can be set between several minutes or hours.
  – External refreshing rate: is application‐specific and could be between several hours or days.

#### 3.1.3 Predictions

Predictions are now an inherent part of modern BMSs. It helps improve the battery performance, utilisation and safety. In real‐world applications, one of the most used predictions is the state of Power (SoP). It estimates the maximum available battery power within a specific time horizon, ensuring that the battery operates within its SOA. In high‐power and high‐dynamic applications such as EVs (including HEVs), the SoP is a key parameter that contributes to improved battery performance and safety. An accurate SoP enables more optimal battery energy utilization and helps prevent overcharging, deep discharging, overheating, and overcurrent events, all of which accelerate battery degradation. Therefore, it should be considered by upcoming regulations. It is usually expressed in W or kW. SoP is limited by several factors, including the battery SOA, its chemistry, the SoH and SoC.^[^ ^78^ ^]^ Therefore, its prediction is challenging. Advancements and current state‐of‐the‐art have been reviewed in the literature.^[^ ^78^, ^79^ ^]^

Propositions for the SoP prediction are made in this section and can be summarized as follows:

- Unit: The W or kW is widely used in the literature.^[^ ^78^ ^]^
- Accuracy: SoP should have a high accuracy, as it is directly correlated to the battery safety (SOA violation). In Ref. [78], the error of reviewed SoP algorithms is reported to be below ±2%. This can serve as orientation.
- Refreshing rate (speed):
  – Internal refreshing rate: It is application‐specific. In the case of highly dynamic applications (EVs, HEVs) or scenarios, the prediction should happen regularly. Interviews in Ref. [34] revealed that stakeholders would appreciate a refreshing rate between 20 and 50 Hz (20–50 ms).
  – External refreshing rate: It is usually less than the internal refreshing rate and should be application‐specific.

### 3.2 Safety and Energy Management

A review of current valid standards and legislations for BMS in Germany revealed multiple standards addressing qualitative requirements for the BMS behavior in critical scenarios. They specify how the BMS should react to ensure a safe battery operation. However, there remain some lacks and improvement potentials. These are summarized in **Table** **5**.

**Table 5:** Lacks or improvement potentials in the current requirements for the features “Safety” and “Energy Management”.

Functions	Aspects	Lacks/ Improvement potentials
Safety	Safety level (ASIL or SIL)	Mandate a safety level in accordance with the applications
Energy management	Performance	Lack of quantitative requirements about the response time for the BMS reactions in safety‐critical scenarios

For example, requirements define the BMS behavior in case of a violated SOA but do not specify the response time *T*_*Res*_. It defines the time range or duration during which the BMS should perform a defined action. *T*_*Res*_ has not yet been specified in the current standards. This could lead to increased safety risks and battery degradation. Propositions for *T*_*Res*_ should consider the severity of the occurred scenario. For instance, in the case of overtemperature, a condition that can pose a serious safety hazard, the BMS must respond immediately to prevent thermal runaway. Similarly, in the event of low insulation resistance, the BMS response time should be short enough to eliminate the risk of electric shock to users. The BMS is typically required to possess real‐time capabilities, enabling it to respond promptly in safety‐critical situations. Real‐time capability refers to the BMS's ability to execute specific tasks within a predefined and deterministic time window, also referred to as the response time *T*_Res_. This is essential for ensuring timely intervention in scenarios such as SOA violations and other errors. Propositions for acceptable bounds of *T*_Res_ in various safety contexts are summarized in **Table** **6**.

**Table 6:** Improvement proposition for the BMS's response time.

Situation	response time	Justification
Overcharge	<1 s	The BMS must interrupt the current for safety reasons
Deep discharge	<1 s	The BMS must react promptly (reduce or interrupt the current) for safety reasons
Overtemperature	<1 s	The BMS must react promptly (reduce/interrupt the current and/or increase the cooling power) for safety reasons
Undertemperature	<1 s	The BMS must react promptly (reduce/interrupt the current and/or increase the heating power) for safety reasons
Low insulation resistance	<100 ms	The BMS must interrupt the battery current promptly, as it could lead to severe hazards for the users

To improve the consistency and interoperability among BMS, future standards should include a requirement regarding their SIL/ASIL level, which BMS should provide for specific applications (refer to *IEC 61508* and *ISO 26262*):

- EVs: At least SIL 2/3 or ASIL‐B/C, considering the safety‐critical nature of traction systems.
- BESSs and mobile phones: At least SIL‐3 or ASIL‐C, as safety hazards in these applications can lead to severe injury or fatality.

Tests and testing procedures should also be included in future standards to ensure a BMS meets these requirements.

### 3.3 Thermal Management

The review of the current requirements for battery thermal management indicated that only high‐temperature SIBs are addressed. Although LIBs are far more widely deployed, their thermal management has not yet been addressed. To bridge this gap, this section proposes improvements based on both qualitative and quantitative requirements. These are summarised in **Table** **7**. Operating LIBs beyond their maximum allowable temperature can lead to severe degradation and even thermal runaways (e.g., fires or explosions). Certain standards mandate the BMS to keep the battery temperature within its SOA without specifying up to what level. This leads to poor implementations in practice, where only a few temperature points are monitored. This is, in certain cases, not enough to promptly detect SOA violations and thermal runaways. Consequently, future standards should mandate thermal management to ensure that the battery temperature, down to the lowest level (cell level), does not exceed its SOA. Furthermore, future standards should include tests and testing procedures to certify that a thermal management system meets these requirements.

**Table 7:** Lacks or improvement potentials in the current requirements for battery thermal management.

Functions	Aspects	Lacks/Improvement potentials
Cooling and heating	Functional safety	Mandate thermal management to ensure the battery remains within the safe operating temperature range
	Performance	Quantitative requirements for the performance of the thermal management system (speed, power, temperature gradient)

Quantitative requirements for the thermal management system could also be included by considering application specifications, such as battery size, expected load profiles, and environmental conditions (e.g., ambient temperature). As a result, specifying global requirements can be a challenging task. Instead, the requirements for the thermal management performance must be tailored to the particular application. Possible performance criteria are listed as follows:

- Accuracy of the temperature control
- Heating and cooling power/speed
- Maximal temperature gradient/uniformity

For example, in BESSs, BMS could be required to maintain cell temperatures within ±3 °C of the target temperature.

### 3.4 Communication and Data Management

Internal and external BMS communications are critical for the safe and reliable operation of battery systems. Communication failures remain a frequent source of BMS malfunctions and overall system failures. Given the vital role of communication, BMS interfaces should comply with high safety integrity levels (SIL or ASIL) to ensure reliability and robustness. Moreover, quantitative performance requirements should be defined, taking into account the communication speed and latency, which are strongly dependent on the specific application. These requirements not only enhance the safety and performance of the BMS but also promote interoperability, which is essential, especially in the context of increasing emphasis on efficient resource utilisation and second‐life battery applications. **Table** **8** summarises the noted lacks and improvement potentials for the BMS communication.

**Table 8:** Lacks or improvement potentials in the current requirements for BMS communication and data management.

Functions	Aspects	Lacks/ Improvement potentials
Internal and external communication	Safety integrity	Lack of mandatory safety levels (SIL/ASIL) for BMS communication
	Performance	Define performance requirements for the BMS communication (speed, latency) based on application needs
	Interoperability	Standardise communication interfaces to enable component reuse (e.g., second‐life)
	Cybersecurity	Missing requirements for data access, control, and transmission
Data recording and storage	Performance	Minimum storage period, refreshing rate or step time

The authors of [76, 80, 81] emphasise the emerging trends in BMS development, including the transition toward cloud‐based BMS architectures, vehicle‐to‐everything (V2X) integration, and broader Internet of Things (IoT) connectivity. These advances will impose more stringent demands on BMS communication, particularly in terms of reliability, robustness, and cybersecurity. In this regard, future standards should increasingly address aspects of information technology (IT) and cybersecurity. A key requirement will be the implementation of mechanisms that ensure protection against unauthorised access to sensitive BMS data.

Another crucial area for standardisation concerns BMS data recording capabilities. Many BMS algorithms operate recursively and require historical battery data, which is typically logged and supplied by the BMS itself. As such, the BMS must be capable of recording operational data over defined periods. However, this requirement introduces challenges related to onboard data storage capacity, which is usually limited due to cost reasons. In addition, regulatory frameworks such as the EU battery passport mandate that certain data, such as SoH, the number of completed equivalent full cycles, energy throughput, fault history, and other operational metrics, must be provided and updated by the BMS and relevant stakeholders. Therefore, it is essential that future standards define clear quantitative requirements regarding the data refreshing rate of measured, estimated, and predicted parameters.

### 3.5 Non‐Functional Requirements

As mentioned in the review section, non‐functional requirements are important, as they can both influence the BMS's hardware and software. Therefore, requirements could be formulated for the BMS hardware and software to enhance their safety and performance.

In mobile applications, the BMS hardware is typically powered directly by the battery it monitors and draws energy from it, gradually reducing its SoC over time. Excessive BMS power consumption can significantly impact the battery autonomy. For instance, a continuous BMS current consumption of 500 mA can fully discharge a 40 Ah battery in just 80 h (approximately 3.33 days), assuming no other loads are connected and no charging occurs. Therefore, it is essential to define and enforce stringent power consumption requirements for BMS hardware, especially in applications where prolonged standby time is usual. Limiting the maximum allowable current draw of the BMS is a practical approach to ensure system efficiency and battery longevity. In practice, BMSs usually have a standby mode, reducing the BMS consumption during the resting period of the battery. In this regard, validation or certification metrics with different levels could be proposed in future standards. In addition to the hardware consumption, requirements could also be addressed to hardware components and performance (e.g., microcontroller, RAM, storage memory), as these indirectly set boundaries, defining the software performance and thus the algorithms that can be used. For instance, the BMS microcontroller unit (MCU) should provide sufficient computational power for the specified algorithms to work in real time. Additionally, interviewees in ref. [34] require a sufficient non‐volatile memory for initializing key parameters and possibly for data recording. An additional requirement for BMS hardware could concern its operational lifetime. Like all electronic components, BMS hardware is subject to ageing processes that degrade its performance over time. However, batteries in applications such as EVs and BESSs are typically expected, due to warranty or insurance conditions, to last at least 8–10 years. Consequently, the BMS must be designed to operate reliably at least over the same period, ensuring consistent monitoring, control, and safety functionality throughout the battery's service lifetime.

BMS algorithms are expected to run under various conditions. Therefore, they should perform reliably also under unexpected conditions. Future standards could also include test and validation procedures for ensuring BMS algorithms are robust before being integrated into real‐life applications. Further requirements are listed in Ref. [34].

In recent years, the topic of second‐life applications for batteries has attracted growing attention, particularly in the context of sustainability and resource optimisation. This trend raises an important question: in battery repurposing, should only the battery pack itself be reused, while the original BMS is discarded? From a sustainability perspective, it is highly desirable to repurpose the BMS along with the battery pack. Reusing the BMS not only reduces electronic waste but also lowers the overall cost and complexity of second‐life systems. However, current BMS designs and practices often hinder reuse due to issues such as software locks, proprietary communication protocols, and a lack of documentation. To address this, future standards could introduce specific requirements for the second‐life usability of BMSs. For example, regulations might mandate that OEMs provide a secure mechanism, such as a manufacturer‐issued unlock key or access token to erase or overwrite the original BMS firmware. This would enable authorized repurposers to deploy their own control algorithms while still utilizing the existing BMS hardware. Such measures would support the development of a circular economy and encourage innovation in battery reuse, particularly in applications such as stationary energy storage, microgrid systems, or low‐power electric mobility.

## 4 Limitations and Future Perspectives

Despite its potential for different stakeholders in the battery sector, the review presented in this paper presents limitations. These should be highlighted to clarify the boundaries of this study and identify areas for further exploration.

### 4.1 Limitations

This review focuses on the German and European regulatory landscape, excluding standards and regulations from other significant countries, such as the United States, China, Japan, and South Korea. As a result, it may miss some localized practices and trends. Furthermore, proprietary or internal company standards were not considered due to their limited public accessibility, although they often represent state‐of‐the‐art implementations. The analysis focuses on LIBs, which are currently dominant in the market, but it does not fully account for the emerging chemistries, such as solid‐state batteries. Additionally, specific requirements and challenges for BMS in second‐life or repurposed applications remain underexplored. However, proposed improvement points may help reduce gaps in requirements, inconsistencies in the real‐world applications, and thus enhance interoperability among batteries from different manufacturers. This can facilitate second‐life applications of batteries.

### 4.2 Future Perspectives

Future research should extend beyond the European context to include other regional standards and regulatory approaches, looking toward a global harmonization. Incorporating insights from proprietary or industrial best practices, where possible, can help bridge the gap between formal regulations and innovative solutions in the field. As the reuse of batteries becomes increasingly relevant, the development of dedicated BMS standards for second‐life applications will be essential. The emergence of wireless and cloud‐connected BMS architectures may pose higher requirements in terms of robust cybersecurity standards and reliable communication protocols.

## 5 Conclusion

This paper provides a comprehensive review of current standards and regulations applicable to BMSs in Germany, with a particular focus on both functional and non‐functional requirements. The analysis reveals that, although a broad array of standards exists, most remain qualitative in nature and lack measurable benchmarks for evaluating BMS performance. To address this gap, the paper proposes concrete improvements, including standardized definitions for SoC and SoH, enhanced accuracy and resolution for key measurements, and quantitative criteria for thermal management, communication, and safety functions.

A central contribution of this work is the systematic mapping of standards to core BMS functionalities, accompanied by concrete proposals aimed at improving consistency, reliability, and interoperability. The paper also highlights the trend toward wBMS, cloud‐based solutions, and IoT‐enabled architectures, emphasizing the need for adaptive and future‐oriented regulations and requirements. Although AI‐enhanced BMS solutions are considerably gaining attention due to their superior performance, the suggested requirements presented in this paper deliberately avoid mandating specific methods. The authors consider the role of standards in defining requirements and objectives, rather than prescribing implementation methods.

While the study focuses on publicly available German and European standards, it does not address proprietary specifications or national regulations from other leading battery markets. Future research should explore these areas to support the development of harmonized international BMS standards. Overall, this paper provides a solid foundation for advancing regulatory efforts and offers guidance to stakeholders aiming to enhance the safety, performance, and interoperability of battery systems. In particular, as second‐life battery applications become increasingly relevant, standardized BMS requirements will be essential to ensure their safe and efficient repurposing.

## Conflict of Interest

The authors declare no conflict of interest.
